# The importance of occupation in the development of the COVID-19 pandemic

**DOI:** 10.5271/sjweh.4094

**Published:** 2023-05-01

**Authors:** Alex Burdorf, Reiner Rugulies

**Affiliations:** Erasmus Medical Center Rotterdam, Department of Public Health and Department of Psychology, Rotterdam, The Netherlands. [email: a.burdorf@erasmusmc.nl]; Department of Public Health, National Research Centre for the Working Environment (NFA),University of Copenhagen, Copenhagen, Denmark. [email: rer@nfa.dk]

In the past three years, we have witnessed the devastating effects of the COVID-19 pandemic, with unprecedented challenges to all aspects of human life worldwide. In the workforce, it rapidly became clear that workers in some jobs were more likely to suffer adverse consequences for morbidity and mortality. In our earlier editorials in the *Scandinavian Journal of Work Environment and Health*, we reviewed emerging evidence, suggesting that well-established socio-economic health inequalities intermingled with occupational risk factors, making it difficult to target the conditions at work that contributed to the transmission of SARS-CoV-2 in working populations (1, 2). As a first priority for the research agenda on COVID-19, we suggested the identification of occupations at higher risk for becoming infected and specific work characteristics that contribute to the risks. Such insights will be immensely valuable for preparedness to threats of future pandemics (2).

Many researchers have addressed this pertinent question with gusto in different populations with different study approaches. In an illustrative example with a population-based approach, Nafilyan and colleagues (3) constructed a register-based cohort study of all 14 million people aged 40–64 years in England for confirmed or suspected COVID-19 death in 2020 across 41 occupational categories. Occupations with the highest age-standardised mortality rates (5–6-fold) were those working as taxi and cab drivers or chauffeurs, workers in elementary occupations, and care workers and home carers. Adjustment for sociodemographic factors attenuated the elevated mortality rates, and further adjustment for living conditions resulted in a residual variance of 20–30% as the best estimate of the maximum contribution of workplace exposure to COVID-19 mortality (3). A comparable registered-based study in Sweden among 4.6 million citizens with an occupation confirmed the highest mortality risk among taxi and bus drivers (4-fold risk), and also pointed towards elevated risk among cleaners and service workers. Likewise, adjustment for socioeconomic factors, such as education and income, greatly reduced the excess risk due to occupation. In contrast, country of birth and income tertile were the strongest socioeconomic determinants of COVID-19 mortality, and these associations were only marginally attenuated by adjustment for occupational groups (4).

In countries with advanced hospital admission registers, occupational risk of COVID-19 related hospital admission can be studied. In Denmark, Bonde et al (5) reported an increased risk for hospitalization due to COVID-19 among healthcare workers, social workers, and a few occupations within transportation compared to office workers. Risk of hospital admission was also increased among spouses of workers in occupations with a high COVID-19 risk (6). The excess risk for hospital admission among healthcare workers, but not among other high-risk occupations, attenuated in the latest COVID-19 waves (7).

Do these studies point towards the conclusions that occupation will at best play only a minor role in COVID-19 mortality? From a methodological point of view, we need to consider whether adjustment for education and income in the association between occupation and COVID-19 mortality is a sound strategy. Since education partly determines working careers and, thus, occupations that people will hold, over-adjustment may be a serious risk. From an exposure point of view, we would preferably measure airborne exposure of SARS-CoV-2 across occupations, and subsequently link levels of exposure across occupations to mortality patterns. Unfortunately, measuring exposure to a coronavirus is still a farfetched dream, although newly developed methods for waste-water surveillance may hold some promise (8).

An alternative to hospitalization and mortality studies is to study SARS-CoV-2 infection rates across occupations. Early reports have shown that this is not a simple task. There are a variety of reasons why people get tested for SARS-CoV-2, such as access at work and employer requirements on testing regimes. Thus, differences in likelihood of being tested may contribute to biased comparisons across occupations (9). Recently, the test-negative design has been advocated as suitable design, eliminating bias through likelihood of being tested by comparing those with a positive test to those with a negative test, thus excluding those who have never been tested from the reference group (10).

In this issue of the *Scandinavian Journal of Work Environment and Health*, the study by Eekhout and colleagues illustrates its usefulness (11). Based on over 200 000 workers with serological SARS-CoV-2 test results, the test-negative design was applied to identify occupations with increased risk for infections, while simultaneously talking into account potential confounders, such as socioeconomic position and household composition. Occupations with high infection rates were quite similar to those reported in the mortality studies described above, with many elementary and manually skilled jobs showing the highest probability to a positive test. The study also applied a job exposure matrix (12) with four work-related factors that could influence the transmission of SARS-CoV-2 at the workplace. Findings showed modestly increased odds of a positive test for these risk factors, and adjustment for several covariates slightly decreased or increased the odds ratios. However, due to substantial collinearity between these work-related risk factors, it was not possible to single out the relative contribution of each factor separately to the infections rates. The magnitude of the observed odd ratios (varying between 1.1 to 1.8) and the prevalence of the work-related risk factors suggest that the proportion of SARS-CoV-2 infections in the general population that is attributable to factors at work will not exceed 30%. This is in line with results of another job-exposure matrix, applied in a French cohort, suggesting that the proportion of COVID-19 cases attributable to work was 20–40% (13).

Why are some occupations at risk and others not? Recent studies have reported in many occupations elevated risks of COVID-19 related morbidity and mortality. The occupational risk factors seem to be interrelated to well-established socioeconomic determinants of health inequalities that are also applicable to COVID-19. The few studies on associations between work-related risk factors and SARS-CoV-2 infection rates provide a rather scattered picture. This is likely partly due to uncertainty about mechanisms of transmission and appropriate mitigation measures (14). Emerging evidence clearly indicates that work plays a role, albeit limited, in the transmission of the coronaviruses and, as such, offers a point of entry for prevention strategies. We must learn from the valuable experiences in the past three years how conditions at the workplace have contributed to the rise and fall of the COVID-19 pandemic.
